# Efficacy analysis of three-year subcutaneous SQ-standardized specific immunotherapy in house dust mite-allergic children with asthma

**DOI:** 10.3892/etm.2014.1469

**Published:** 2014-01-02

**Authors:** YU HUI, LING LI, JUN QIAN, YUN GUO, XILIAN ZHANG, XIAOJUAN ZHANG

**Affiliations:** Department of Respiratory Medicine, Wuxi Children’s Hospital Affiliated to Nanjing Medical University, Wuxi, Jiangsu 214023, P.R. China

**Keywords:** SQ-standardized specific immunotherapy, inhaled corticosteroids, asthma, house dust mite, children

## Abstract

The present study aimed to evaluate the efficacy of three-year subcutaneous SQ-standardized specific immunotherapy (SCIT) in house dust mite (HDM)-allergic children with asthma. Ninety children with allergic asthma to HDMs, with or without allergic rhinitis, were randomly divided into two groups, the treatment group and the control group. The treatment group received SCIT combined with standardized glucocorticoid management and the control group received standardized glucocorticoid management alone for a period of three years. The mean daily dose of inhaled corticosteroids (ICSs), a four-week diary recording the symptom scores of asthma, peak expiratory flow (PEF) measurements, skin prick test results and serum immunoglobulin E (IgE) levels were assessed prior to treatment and following one, two and three years of treatment. The median dose of ICS was reduced in the treatment group after two and three years of treatment compared with that of the control group. After three years of treatment, the discontinuation percentage of ICS in the treatment group was higher than that in the control group. The treatment group demonstrated significantly reduced daytime and night-time asthmatic symptom scores, increased PEF values and reduced serum IgE levels after two and three years of treatment compared with those in the control group (P<0.05). In conclusion, three-year SCIT treatment combined with ICS is an effective immunotherapy for children with allergic asthma and resulted in a reduction of the required ICS dose.

## Introduction

Asthma is one of the most common chronic diseases in pediatric medicine. It is estimated that the annual morbidity and mortality rates have been increasing in recent years worldwide. According to a report by the World Health Organization, >80% of asthma in children results from an allergic reaction in which the house dust mite (HDM) is the major pathogen (1). Inhaled corticosteroids (ICSs) and specific immunotherapy (SIT) are widely used inflammatory treatments for controlling the symptoms of asthma (2). Although ICSs remain the recommended agents for asthma control, the use of an ICS alone is not beneficial due to the risk of side-effects, including oropharyngeal candidiasis, trachyphonia and cough. Recently, a number of studies have demonstrated that combined ICS and SIT therapies may alleviate the symptoms of asthma and nasal allergies, and reduce the required dose of medications (3,4). It is well known that the administration of SIT by subcutaneous injection is beneficial to patients with asthma and its complications. Subcutaneous SIT may improve the prognosis of asthma and allergic rhinitis and enable the daily dose of glucocorticoid to be reduced (5,6). Systemic pre-clinical investigations regarding the long-term effects of this combination therapy are lacking, and the effects of combined SIT and ICS administration have only been determined from patient experience. Moreover, the appropriate dosage, clinical observations, potential side-effects and outcome of ICS combined with subcutaneous SQ-standardized specific immunotherapy (SCIT) in the treatment of children with asthma remain unclear. In the present study, a systemic three-year evaluation was performed to compare the efficacy of standardized glucocorticoid management with or without SCIT in the treatment of children with HDM allergic asthma.

## Subjects and methods

### Patients

Ninety asthmatic children (with or without allergic rhinitis) with a mild to moderate HDM allergy (aged, 5–14 years) were recruited from January 2009 to December 2009 at Wuxi Children’s Hospital affiliated to Nanjing Medical University (Wuxi, China). This was a randomized, double-blind, placebo-controlled study. The patients were separated into two groups: The treatment group (n=45; males, 24 and females, 21) and the control group (n=45; males, 22 and females, 23). Patients in the treatment group received Alutard SQ (*Dermatophagoides pteronyssinus*; ALK-Abelló, Hørsholm, Denmark) SCIT combined with standardized management (ICS) for 36 months. The patients in the control group were also treated with a desensitization vaccine. The standardized management was administered with the vaccine kit (desensitization vaccine, ALK-Abelló). This study was conducted in accordance with the Declaration of Helsinki and with approval from the ethics committee at Wuxi Children’s Hospital affiliated to Nanjing Medical University. The legal guardians of all patients were informed of the treatment and written informed consent was obtained from the participants and (or) their legal guardians.

Inclusion criteria: i) Asthma diagnosis followed the diagnostic criteria established by the National Pediatric Asthma Group (7); ii) patients aged between 5–14 years (including males and females); iii) patients showed mild to moderate allergic asthma with or without allergic rhinitis and a forced expiratory volume in 1 sec (FEV1) of ≥70% of the normal value; iv) patients tested positive in the skin prick test (SPT) and had a urticaria skin index (SI) of ≥0.5 (++) and/or tested positive for allergen-specific IgE in the serum; and v) patients required ICS treatment to control the symptoms of asthma.

Exclusion criteria: i) Patients displayed a FEV1 of <70% of the normal value; ii) patients diagnosed with severe asthma; iii) patients used ICS to control asthma during this study; iv) patients treated with a daily dose of ICS >800 g beclomethasone for 15 days or patients routinely administered prednisone orally; v) patients who were receiving treatment with other medicines, such as leukotriene modifiers and long-acting β agonists to control asthma; vi) patients commonly suffering from respiratory tract infection, acute sinusitis or acute otitis media; vii) patients treated for HDM or other allergens in the previous five years; viii) patients previously diagnosed with heart, lung, liver, kidney or blood diseases; ix) patients receiving treatment with receptor blockers; and x) patients who had received previous immunotherapy with immunosuppressants for immunodeficiency.

### Treatment

All patients were treated with the standardized management for HDM. The SCIT treatment was initiated at a dosage of 20 U/ml, and was continued weekly with an increase in the dosage each week; the dosages were 20, 40, 80, 200, 400, 800, 2,000, 4,000, 8,000, 10,000, 20,000, 40,000, 60,000, 80,000 and 100,000 U/ml, respectively. Following the 15 treatments, patients received maintenance treatment in weeks 17, 21, 27, 33, 39, 45 and 51 with a dose of 100,000 U/ml. The SCIT treatment was discontinued following the final treatment at week 51 according to the symptoms of asthma in the patients and the clinical experience of agent administration.

### Assessments

To evaluate the efficiency of the combined immunotherapy, there were 10 check-points during the treatment period at which certain parameters were monitored. The first check-point was prior to treatment and the remaining check-points were following the start of treatment at weeks 1, 15, 27, 39, 51, 75, 99, 123 and 147. Five parameters were monitored, including the dose of ICS, asthma symptom scores, peak expiratory flow (PEF) levels, SPT results and serum IgE levels.

### Dose of ICS

Patients were treated with ICS according to the findings of a pediatric asthma control trial (5). The therapeutic protocol was revised every 1–3 months. When the symptoms of asthma had been controlled for three months, the dose of ICS was reduced. Complete withdrawal of the ICS was considered if no asthma symptoms had been observed in the patient for six months. The glucocorticoids inhaled were budesonide (AstraZeneca, North Ryde, NSW, Australia) and fluticasone propionate (Glaxo Wellcome, Brentford, UK).

### Asthma symptom scores

The symptoms of asthma were scored at daytime and night-time as follows: Daytime score: 0, no symptoms; 1, mild symptoms appear intermittently; 2, moderate symptoms frequently appear; 3, enduring symptoms affecting routine activity. Night-time score: 0, no symptoms; 1, discomfort when waking up once or waking up early; 2, discomfort when waking up more than once; 3, discomfort when waking up at night frequently but able to fall asleep; 4, sleeplessness.

### PEF

The evaluation of PEF was performed using a spirometer (AS-407; Minato Medical Science Co., Ltd., Osaka, Japan). To measure the PEF levels of a patient, the indicator was adjusted to ‘0’ and the instument was steadied. The patient was required to breathe deeply and then blow strongly into the instrument for minimal time. This evaluation was performed three times and the highest PEF level was recorded.

### SPT

For the SPT, the standard prick antigen ALK histamine dihydrochloride (ALK, Copenhagen, Denmark) was used as a positive control and saline was defined as a negative control (8). The diameter of the wheal and red spot (S) was calculated using the following formula: S = (d + D)/2, where d is the smallest transverse diameter and D is the largest transverse diameter. D and d crossed at right angles. A positive result was achieved if the wheal diameter (S) was larger than that of the negative control by 3 mm. The SI of the patients was calculated using the following formula: SI = diameter of the allergen-induced wheal/diameter of the histamine-induced wheal. The SI was graded as follows: Normal, ‘0’ = negative; grade I, ‘+’ = SI<0.5; grade II, ‘++’=0.5≤SI<1.0; grade III, ‘+++’=1.0≤SI<2.0; grade IV, ‘++++’=2.0≤SI.

### Serum IgE analysis

The serum IgE levels of HDMs in the two groups were measured using a specific house mite test kit (Dr. Fooke-Achterrath Laboratorien GmbH, Neuss, German) and the UniCAP immune detection system (Pharmacia and Upjohn, Stockholm, Sweden). The reference value of the test result was 0–0.35 kU_A_/l for a normal result.

### Statistical analysis

All data are expressed as the mean ± standard deviation and were analyzed with SPSS software, version 11.5 (SPSS, Inc., Chicago, IL, USA). Comparisons within groups and among the groups were analyzed by nonparametric tests for multiple samples and t- and q-tests. P<0.05 was considered to indicate a statistically significant difference.

## Results

### General evaluation

In the treatment group, there were 45 patients (24 males and 21 females) and the average age was 10.1±2.2 years. In the control group there were 45 patients (22 males, and 23 females) and the average age was 9.8±1.5 years. There were no statistical differences between the two groups regarding the baseline parameters, such as age, gender, duration of asthma, dose of ICS, SPT results and levels of PEF and serum IgE (Table I). In the treatment group, five patients were withdrawn (three males and two females) within the 12 months following the first injection. In three of these cases, local indurations remained following injection. One patient experienced pharyngeal discomfort and coughing and another displayed a systemic allergic reaction. Twenty-four months following the first injection, a further two patients were withdrawn from the study (one of each gender); one demonstrated urticaria and the other experienced coughing 15–30 min after injection. By 36 months following the initial injection, two more male patients were withdrawn; one left the study and the other experienced a tight chest 30 min after injection that was not alleviated by dose reduction. In the control group, four patients (three males and one female) were withdrawn within the first 12 months. All four patients experienced no improvement of asthma symptoms. At 24 months, two further patients were withdrawn (one of each gender) for personal reasons. At 36 months, an additional male patient left the study.

### Dose of ICS

**Figure 1:**
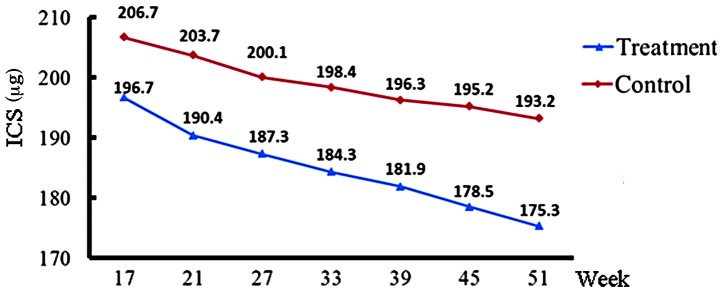
ICS dose of the treatment and control groups at weeks 17, 21, 27, 33, 39, 45 and 51. ICS, inhaled corticosteroid.

The ICS doses in the treatment and control groups decreased gradually with time. The ICS doses in the treatment and control groups in the first year are shown in Fig. 1. The dose of ICS in the treatment group was significantly lower than that in the control group in the second (P=0.015) and third years (P=0.027; Table II). At the end of the third year, 13 cases in the treatment group and nine cases in the control group had ceased ICS treatment. The ICS discontinuation rate in the treatment group (28.9%) was significantly higher than that in the control group (20.0%) (Z=−2.327, P=0.020).

### Evaluation of asthma symptoms

Daytime and night-time asthma symptom scores were recorded. The scores of the treatment and control groups declined from baseline during the course of treatment. Consistent with the ICS dose, the asthma symptom scores of the treatment group were significantly lower each year compared with those of the control group (P<0.05; Table III).

### PEF evaluation

The accumulation of standardized allergen extracts of HDM during treatment resulted in a significantly increased PEF value compared with that prior to injection (P<0.05). The increase in PEF was more marked in the second and third years than that of the first year (Table IV).

### SPT evaluation

The SPT results remained essentially unchanged at the annual re-assessments. No differences between the two groups were identified.

### Serum IgE levels

The serum IgE levels were significantly reduced compared with baseline levels at the end of the third year in the treatment group (P<0.01), but not in the control group (P=0.241). For this phenotype, the serum IgE level was significantly downregulated by combined therapy. This effect was only observed following at least three years of treatment; no significant differences were identified in in the first (P=0.897) and second (P=0.665) years. No significant differences were observed within the control group (P=0.241; Table V).

### Adverse reactions

Adverse reactions following injection were monitored and it was identified that 203 out of the 1,735 injections were associated with an adverse reaction. One of the 203 injections was a systemic adverse reaction and the remainders were local adverse reactions.

## Discussion

The primary pathogenesis of asthma is an immune reaction in which HDM is the most common pathogen. ICSs, anti-allergic agents and support treatments are well-known clinical asthma therapies. However, the long-term use of ICSs is not beneficial due to the risk of side-effects. SCIT may improve allergic diseases, including asthma (9,10). The present study aimed to systemically evaluate the effects of SIT in children. This study was an open clinical observation following the intention-to-treat principle, in which parallel controls and self-controls were established (9).

This study demonstrated that ICS treatment improved asthma symptom scores, serum IgE levels and PEF values with or without immunotherapy. However, a comparison of the treatment and control groups showed certain differences. The daytime and night-time asthma symptom scores in the treatment group were significantly lower than those of the control group. In addition, the PEF values and serum IgE levels were lower in the treatment group compared with those of the control group, indicating that specific immunotherapy was effective in the treatment of children with asthma. The dose of ICS also decreased each year in the two groups. However, the reduction was most evident in the treatment group in the second and third years. Following the third year of treatment, the rate of ICS discontinuation in the treatment group was significantly higher than that in the control group. The serum IgE levels in the treatment group were lower than those in the control group. These results suggest that long-term SCIT may alleviate asthma symptoms and reduce the required dose of ICS. Previous studies have demonstrated that subcutaneous injection immunotherapy is effective in the treatment of allergic rhinitis and asthma, which may improve the symptom scores by >40% (11–15). A previous study indicated that SCIT treatment may alleviate the clinical symptoms of allergic rhinitis as early as 6 weeks following the initiation of treatment (16). The present three-year retrospective study on the effects of SIT in the treatment of HDM-allergic asthmatic children showed that SCIT may improve lung function and the clinical symptoms of asthma. It may also reduce the number of asthmatic attacks and enable the ICS dose to be reduced.

In the present study, the total number of injections was 1,735. Among them, 203 injections resulted in adverse reactions. One case displayed a systemic adverse reaction and the remaining cases showed local adverse reactions. Similar to previous studies (17,18), the local adverse reactions in the present study were manifested as local induration, induced cough and urticaria. The rate of adverse reactions was 11.7%. The incidence rate of adverse reactions in asthma-SIT was previously observed to be 5–33% (19). Those adverse reactions occurred in the dose-increasing and maintenance periods, which is also consistent with a previous study (20).

At present, the recommended course of treatment with SIT is 3–5 years and the effects may last for a long time even once treatment has finished (21). However, there is no standard course of treatment for asthma and there is a 0–55% relapse frequency rate. Different courses of treatment and the diversity of allergens may affect the length of clinical remission following drug withdrawal (9). The efficacy of SCIT treatment may also vary according to the severity of the disease and the purity of the extracts. Therefore, the most effective SCIT treatment should be an individualized treatment and its standards should be determined by clinical studies.

In conclusion, this study demonstrated that SCIT is effective and safe for the treatment of children with allergic asthma, alleviates asthma symptoms and reduces the required ICS dose.

## Figures and Tables

**Table I:** General patient data.

Characteristic	Treatment group	Control group	Z- or t-test	P-value
Gender				
Male	24	22		
Female	21	23	Z=−1.026	0.305
Age (years)	10.1±2.2	9.8±1.5	t=0.542	0.590
Course (years)	3.5±1.4	3.4±0.9	t=0.217	0.829
Asthma score				
Day	2.8±0.7	2.8±0.5	t=0.094	0.925
Night	1.8±0.4	1.9±0.4	t=−1.139	0.259
ICS (μg)	196.7±65.6	206.7±45.0	t=−1.775	0.081
Serum IgE (kU_A_/l)	91.4±29.1	90.9±19.2	t=0.074	0.941
PEF value (%)	63.3±5.4	62.3±5.1	t=0.074	0.941
SPT	1.2±0.5	1.3±0.5	t=−0.629	0.532

ICS, inhaled corticosteroid; IgE, immunoglobulin E; PEF, peak expiratory flow; SPT, skin prick test. ICS doses are presented as budesonide equivalents. Measurement data are the mean ± standard deviation.

**Table II:** ICS doses in the treatment and control groups.

Variable	Baseline (μg)	Year 1 (μg)	Year 2 (μg)	Year 3 (μg)
Treatment group	196.7±65.6	170.8±64.4	115.0±54.1	71.3±53.8
Control group	206.7±45.0	190.4±46.8	147.9±47.0	101.3±48.5
*t*	−0.689	−1.346	−2.516	−2.269
P-value	0.494	0.183	0.015	0.027

Intragroup comparison of the treatment group X^2^=89.709, P=0; and the control group X^2^=88.349, P=0. ICS, inhaled corticosteroid.

**Table III:** Asthma symptom scores of the treatment and control groups.

	Baseline		Year 1		Year 2		Year 3	
				
Variable	Day	Night	Day	Night	Day	Night	Day	Night
Treatment group	2.8±0.7	1.8±0.4	2.0±0.7	1.1±0.4	1.1±0.7	0.8±0.3	0.7±0.5	0.4±0.3
Control group	2.8±0.5	1.9±0.4	2.5±0.6	1.5±0.3	1.6±0.6	1.2±0.3	1.0±0.5	0.7±0.3
t-value	0.094	1.139	1.945	1.805	2.064	2.027	2.206	2.365
P-value	0.925	0.259	0.013	0.024	0.012	0.011	0.009	0.007

**Table IV:** PEF results of the treatment and control groups (l/min).

Variable	Baseline	Year 1	Year 2	Year 3
Treatment group	63.3±5.4	72.5±6.3	87.4±9.2	91.3±5.8
Control group	62.3±5.1	69.4±4.8	73.5±5.1	81.6±4.5
t-value	0.941	1.346	2.324	2.769
P-value	0.074	0.063	0.018	0.007

PEF, peak expiratory flow.

**Table V:** Serum IgE levels in the treatment and control groups (kU_A_/l).

Variable	Baseline	Year 1	Year 2	Year 3
Treatment group	91.4±29.1	85.3±18.2	80.4±14.2	77.6±26.4
Control group	92.6±24.5	92.1±18.8	90.3±25.6	90.8±20.5
t-value	1.846	0.818	2.582	3.147
P-value	0.092	0.073	0.024	0.003

IgE, immunoglobulin E.

## References

[1] Carrard A, Pichler C (2012). House dust mite allergy. Ther Umsch.

[2] Barnes PJ (2012). New drugs for asthma. Semin Respir Crit Care Med.

[3] Wang CM, Chuang JJ (2013). Effect of mite allergen immunotherapy on the altered phenotype of dendritic cells in allergic asthmatic children. Ann Allergy Asthma Immunol.

[4] Maazi H, Shirinbak S, Willart M (2012). Contribution of regulatory T cells to alleviation of experimental allergic asthma after specific immunotherapy. Clin Exp Allergy.

[5] Eifan AO, Akkoc T, Yildiz A, Keles S, Ozdemir C, Bahceciler NN, Barlan IB (2010). Clinical efficacy and immunological mechanisms of sublingual and subcutaneous immunotherapy in asthmatic/rhinitis children sensitized to house dust mite: an open randomized controlled trial. Clin Exp Allergy.

[6] Blumberga G, Groes L, Dahl R (2011). SQ-standardized house dust mite immunotherapy as an immunomodulatory treatment in patients with asthma. Allergy.

[7] The Breathing Group of Pediatric Academy affiliated to Chinese Medical Association and the Editorial Board of Chinese Journal of Pediatrics affiliated to Chinese Medical Association (2004). The routine for prevention and treatment of bronchial asthma in children (for trial). Zhonghua Er Ke Za Zhi.

[8] Yang BZ, Chen HB, Hou JM (2011). Clinical analysis on 520 children with bronchial asthma of allergens by skin prick test. Journal of North Sichuan Medical College.

[9] Akdis CA, Akdis M (2011). Mechanisms of allergen-specific immunotherapy. J Allergy Clin Immunol.

[10] Han J, Huang Y, Wang MK (2011). Influence of specific immunotherapy on *Dermatophagoides pteronyssinus*-specific IgG4 and pulmonary function in children suffering from allergic asthma. Journal of Third Military Medical University.

[11] Cox L, Calderón M, Pfaar O (2012). Subcutaneous allergen immunotherapy for allergic disease: examining efficacy, safety and cost-effectiveness of current and novel formulations. Immunotherapy.

[12] Gonzales M, Fratianni C, Mamillapali C, Khardori R (2012). Immunotherapy in miscellaneous medical disorders Graves ophthalmopathy, asthma, and regional painful syndrome. Med Clin North Am.

[13] Trebuchon F, David M, Demoly P (2012). Medical management and sublingual immunotherapy practices in patients with house dust mite-induced respiratory allergy: a retrospective, observational study. Int J Immunopathol Pharmacol.

[14] Hedlin G, van Hage M (2012). The role of immunotherapy in the management of childhood asthma. Ther Adv Respir Dis.

[15] La Rosa M, Lionetti E, Leonardi S (2011). Specific immunotherapy in children: the evidence. Int J Immunopathol Pharmacol.

[16] Stelmach I, Sobocińska A, Majak P, Smejda K, Jerzyńska J, Stelmach W (2012). Comparison of the long-term efficacy of 3- and 5-year house dust mite allergen immunotherapy. Ann Allergy Asthma Immunol.

[17] Eifan AO, Shamji MH, Durham SR (2011). Long-term clinical and immunological effects of allergen immunotherapy. Curr Opin Allergy Clin Immunol.

[18] Klimek L, Mewes T, Wolf H, Hansen I, Schnitker J, Mann WJ (2005). The effects of short-term immunotherapy using molecular standardized grass and rye allergens compared with symptomatic drug treatment on rhinoconjunctivitis symptoms, skin sensitivity, and specific nasal reactivity. Otolaryngol Head Neck Surg.

[19] Creticos PS (2000). The consideration of immunotherapy in the treatment of allergic asthma. J Allergy Clin Immunol.

[20] Frew AJ (2003). 25. Immunotherapy of allergic disease. J Allergy Clin Immunol.

[21] Mellerup MT, Hahn GW, Poulsen LK, Malling H (2000). Safety of allergen-specific immunotherapy. Relation between dosage regimen, allergen extract, disease and systemic side-effects during induction treatment. Clin Exp Allergy.

